# The validation of short eating disorder, body dysmorphia, and Weight Bias Internalisation Scales among UK adults

**DOI:** 10.1186/s40337-024-01095-9

**Published:** 2024-09-09

**Authors:** Dorottya Lantos, Darío Moreno-Agostino, Lasana T. Harris, George Ploubidis, Lucy Haselden, Emla Fitzsimons

**Affiliations:** 1 UTS Business School, University of Technology Sydney, Ultimo, Australia; 2 Centre for Longitudinal Studies, Social Research Institute, UCL, London, UK; 3 Department of Experimental Psychology, UCL, London, UK; 4 ESRC Centre for Society and Mental Health, King’s College London, London, UK

**Keywords:** Measurement, Eating disorder, Body dysmorphia, Weight bias internalisation, Questionnaire optimisation

## Abstract

**Background:**

When collecting data from human participants, it is often important to minimise the length of questionnaire-based measures. This makes it possible to ensure that the data collection is as engaging as possible, while it also reduces response burden, which may protect data quality. Brevity is especially important when assessing eating disorders and related phenomena, as minimising questions pertaining to shame-ridden, unpleasant experiences may in turn minimise any negative affect experienced whilst responding.

**Methods:**

We relied on item response theory to shorten three eating disorder and body dysmorphia measures, while aiming to ensure that the information assessed by the scales remained as close to that assessed by the original scales as possible. We further tested measurement invariance, correlations among different versions of the same scales as well as different measures, and explored additional properties of each scale, including their internal consistency. Additionally, we explored the performance of the 3-item version of the modified Weight Bias Internalisation Scale and compared it to that of the 11-item version of the scale.

**Results:**

We introduce a 5-item version of the Eating Disorder Examination Questionnaire, a 3-item version of the SCOFF questionnaire, and a 3-item version of the Dysmorphic Concern Questionnaire. The results revealed that, across a sample of UK adults (*N* = 987, ages 18–86, *M* = 45.21), the short scales had a reasonably good fit. Significant positive correlations between the longer and shorter versions of the scales and their significant positive, albeit somewhat weaker correlations to other, related measures support their convergent and discriminant validity. The results followed a similar pattern across the young adult subsample (*N* = 375, ages 18–39, *M* = 28.56).

**Conclusions:**

These results indicate that the short forms of the tested scales may perform similarly to the full versions.

**Supplementary Information:**

The online version contains supplementary material available at 10.1186/s40337-024-01095-9.

## Abstract (plain language summary)

This manuscript introduces short versions of existing measures of eating disorders and body dysmorphia, specifically the *Eating Disorder Examination Questionnaire*, the *SCOFF Questionnaire*, and the *Dysmorphic Concern Questionnaire.* We further investigate the properties of the recently introduced 3-item short version of the modified *Weight Bias Internalisation Scale.* Across analyses including measurement invariance testing and bivariate correlations aiming to assess convergent and discriminant validity, we find support that the short scales may perform similarly to their longer versions. These short scales may contribute in meaningful ways to research where the brevity of questionnaire-type measures may make a difference by contributing to data quality.

**Supplementary Information:**

The online version contains supplementary material available at 10.1186/s40337-024-01095-9.

## Background

The time participants volunteer to partake in research is invaluable. Ensuring that participants spend this time in a meaningful way and no unnecessary time is granted is not only an ethical priority, but also a way to obtain high quality data [1, 2]. Questionnaire-type measures are among the most often used methods for data collection in the psychological and social sciences [3, 4]. Historically, such scales have been designed to capture a given construct in an in-depth manner, often resulting in a long series of items, taking a long time to complete. More recently, advances in psychometrics have revealed that fewer items are in many cases sufficient to capture the same underlying construct, without losing meaningful information [5, 6].

The amount of time taken to complete questionnaires is an especially important objective whilst designing test packages for longitudinal cohort studies. In preparation for the upcoming 2023 data sweep of the Millennium Cohort Study (MCS, 7,8), which has followed the lives of nearly 19,000 UK individuals born in 2000–2001, we aimed to optimise selected eating disorder and body dysmorphia scales among a sample of UK adults. The analyses presented here complement our recent analyses performed with the aim of comparing and optimising measures of depression, anxiety, and psychological distress in preparation for the same MCS data sweep [9]. Specifically, here we examined the properties of the 12-item short version of the Eating Disorder Examination Questionnaire (EDE-QS, 10), the 5-item SCOFF questionnaire [11], the 7-item Dysmorphic Concern Questionnaire (DCQ, 12), and the 11-item and 3-item versions of the modified Weight Bias Internalisation Scale (WBIS, 13,14).

Our aim was to ensure that these widely used scales can be administered in as little time as possible, whilst capturing similar variance and information to their original versions. Experiences of eating disorders and body dysmorphia may be highly unpleasant and shame-ridden [15–17]. Thus, asking a limited number of questions regarding such experiences may be especially important in ensuring that participants are exposed to as little amount of stress as possible, without compromising data quality. We further tested the measurement invariance of these self-report scales in order to ensure that they can be used across cohorts, enabling measurement harmonisation and thus facilitating cross-cohort comparisons [18, 19].

### Optimising questionnaires

#### Brevity

Questionnaire-based measures historically tended to comprise many, often dozens of items. This was driven by the intention to truly capture an underlying construct as accurately as possible. However, using such measures may be counterproductive in some cases, as studies which last too long also compromise data quality [2]. Often cited causes for this include boredom effects (i.e., participants’ performance/attention decreases as they become bored and lose interest), response burden (i.e., the effort required to complete questionnaire, which increases as the length of the questionnaire increases), and fatigue (i.e., participants’ performance/attention decreases as they become tired). Longer scales are additionally more likely to result in missing data. One of our aims in this study is to optimise self-report questionnaires for brevity.

#### Bias

Another key issue with questionnaires is potential bias. Several factors may influence the way in which people perceive certain questions, including cultural differences or other differences related to age, including the historical time during which one grew up, etc. [20]. Such bias may lead individuals to interpret questions differently, which ultimately may lead to different constructs being assessed by the same scale [21–23]. Thus, in this study we further assess measurement invariance across sex and age groups.

### Selecting and developing measures for cohort studies

Longitudinal birth cohort studies follow a cohort of participants born around the same time. Such designs allow researchers the opportunity to study the effects of social, economic, and environmental factors on key outcomes across the lifespan [24, 25]. Several birth cohort studies conducted throughout the past decade in the UK are currently still running, including cohorts born in 1946, 1958, 1970, 2000/01. An important factor when selecting and developing measures for inclusion in birth cohort studies is brevity. Brevity contributes to ensuring that participants in longitudinal studies remain engaged and minimises attrition. One way to do this is to optimise scales by minimising their number of items. However, it must be ensured that the included scales are valid and reliable. In addition, it is important to ensure that all measures assess the same construct across different groups, such as across sex or age groups in a population [18, 19]. This further facilitates the comparison across studies, including cross-cohort comparisons.

### Overview of the study

Using an online survey, we explored the properties of existing self-report measures of eating disorders, body dysmorphia, and weight bias internalisation among UK adults. We aimed to optimise selected measures by reducing the number of items which participants are required to respond to. We further examined the same characteristics among only the young adult subsample (18–39 years) and ensured that the optimised short versions of each scale exhibit similar properties in the young adult sample and the full sample. In preparation for the next MCS [7, 8] data sweep, this age group is of special interest. To gather data of the highest possible quality, keeping in mind the limited availability of survey time, we aim to inform the selection of self-report questionnaires for use in the upcoming data sweep (age 22, 2023) with the results presented here.

More specifically, our aim was to find a short set of items that correlate highly with longer widely used scales, but which are less time-consuming to complete. We have tried to shorten the scales based on multiple factors: retaining the maximum amount of information across different levels of the underlying construct, thinking of the general (non-clinical) population, and focusing on reducing participant burden. We have assessed whether these shorter measures may rank-order the participants in a similar way as the longer versions. While undoubtedly there is a loss of granularity with the shortening of scales, data quality may be, overall, be improved this way if, for example, these scales are to be embedded in lengthy questionnaires. Under such circumstances, reducing participant burden is especially important as it may lead to a lack of attention, disengagement, or missing data, among others. Thus, while shorter scales do not necessarily mean better scales, there may certainly be cases where shorter options are better at meeting the researchers’ aims.

As the analyses presented here additionally allow us to optimise these same scales across UK adults of all ages, we aim to inform other researchers who may be conducting studies in this population. We tested measurement invariance to ensure that the scales tested here assessed the same constructs across sex and age groups. The online survey included additional measures of depression, anxiety, and psychological distress as well, which are explored in detail elsewhere [9]. The study was preregistered (https://osf.io/bk9xs)^1^. Ethical approval was obtained from the Ethics Committee of University College London. All data and syntax files are available via OSF (https://osf.io/vg4a9/).

## Method

### Participants

A sample of 1,068 UK adults started the survey. The sample was recruited via Prolific (www.prolific.com) to closely mimic one that is representative of the UK population. To recruit a sample that approximates representativeness, Prolific uses data from the UK Office of National Statistics, and matches participants to the national population as closely as possible on age, gender, and ethnicity. We removed the data of 8 participants who gave consent to partaking but did not consent to the storage of their data, as well as 40 participants who only filled in the consent form and nothing else. We excluded a further 33 participants from data analysis due to incorrect responses to (one or both) attention check questions (e.g., Please select agree). The final sample consisted of 987 participants (463 males, 505 females, 2 participants indicated that they did not wish to share their sex^2^), ages 18–86, *M* = 45.21, *SD* = 15.61. Seventeen participants only partially completed the survey, and their demographics details were thus missing. Participants were recruited via Prolific Academic and reimbursed £7.50 for their time. Across some of the analyses we were interested primarily in the responses of young adults, and hence completed them by including only the 375 participants who were aged 18–39 (*M* = 28.56, *SD* = 6.39, 184 males, 191 females).

### Procedure

Data was collected as part of a larger project in November, 2021 (see for further details: [9]. We created an online survey using Qualtrics software. Participants were first presented with an informed consent form and information sheet detailing their tasks throughout the study. They next completed several psychometric questionnaires, including measures focusing on the assessment of eating disorders and body dysmorphia described below. All scales were presented in a randomized order across participants. Finally, participants responded to demographic questions (sex, gender, age, ethnicity), were debriefed and thanked for their time.

### Measures

***Eating disorders*** were assessed using the 12-item short version of the EDE-QS [10], the 5-item SCOFF questionnaire [11], and the 22-item eating disorder diagnostic scale (EDDS, 23,24).

The EDE-QS [10] was completed by 972 participants. Participants responded to 10 items of the EDE-QS (e.g., On how many of the past 7 days have you had a definite fear that you might gain weight? ) on a 4-point scale with response options 0 = 0 days, 1 = 1–2 days, 2 = 3–5 days, 3 = 6–7 days; and to two items (e.g., Over the past 7 days, how dissatisfied have you been with your weight or shape? ) on a 4-point scale with response options 0 = not at all, 1 = slightly, 2 = moderately, 3 = markedly. Participants’ responses were summed, with higher scores indicating an increased presence of characteristics of eating disorders.

The SCOFF [11] was completed by 975 participants. Participants completed 5 items of the questionnaire (e.g., Do you make yourself sick because you feel uncomfortably full? ) using binary yes/no responses. We scored ‘yes’ responses as 1 and ‘no’ responses as 0, and summed participants’ answers, with higher scores indicating a greater likelihood for the presence of eating disorders.

The EDDS [26, 27] was completed by 974 participants. The 22 items which participants completed included a variety of response methods, e.g., questions asked participants to enter their weight and height, to respond to binary questions with yes/no responses (e.g., During the times when you ate an unusually large amount of food, did you experience a loss of control (feel you couldn’t stop eating or control what or how much you were eating)? ), or to respond to 15 point scales (e.g., How many times per week on average over the past 3 months have you made yourself vomit to prevent weight gain or counteract the effects of eating, with response options between 0 and 14), among others. We used existing code [27] to calculate index scores (raw eating disorder composite score and Z-transformed eating disorder composite score) based on participants’ responses, where higher scores indicate a greater likelihood for the presence of eating disorders. Note that as a diagnostic tool this scale corresponds directly to the DSM-IV rather than the DSM-V diagnostic criteria of eating disorders.

***Body dysmorphia*** was assessed using the 4-item body dysmorphic disorder questionnaire (BDDQ, 25) and the 7-item DCQ [12]. The BDDQ [28] was completed by 997 participants. This scale is made up of four core questions, where each question is presented based on participants’ previous responses (e.g., the question ‘Is your main concern with how you look that you aren’t thin enough or that you might get too fat?’ is only presented if a participant responds ‘yes’ to the question ‘Are you worried about how you look?’). This scale functions as a diagnostic tool for eating disorders. Following the scoring guidelines, we coded participants either as being at risk of an eating disorder (coded 1, overall sample: *N* = 183 out of 987; young adults: *N* = 109 out of 375) or not (coded 0).

The DCQ [12] was completed by 977 participants. Participants responded to the 7 items of the DCQ (e.g., Have you ever been very concerned about some aspect of your physical appearance? ) on a 4-point scale with response options 0 = not at all, 1 = same as most people, 2 = more than most people, 3 = much more than most people. Participants’ responses were summed, with higher scores indicating increased body dysmorphia.

***Weight bias internalisation*** was assessed using the 11-item [14] and 3-item [13] versions of the WBIS. The scales were completed by 978 participants. Participants responded to the items (e.g., I hate myself for my weight) on a 7-point Likert scale with response options ranging from 1 = strongly disagree to 7 = strongly agree. Participants’ responses on selected items were reverse scored and all scores were summed in a way that higher scores reflect increased weight bias internalisation.

***Depression***,*** anxiety***,*** and psychological distress*** were also assessed as part of the survey, though these scales are examined in detail elsewhere [9]. The 10-item K10 scale and the 6-item K6 scale embedded in it [29], the 9-item version of the Malaise Inventory [30, 31], the PHQ-9 [32, 33], PHQ-2 [34], GAD-7 [35], and GAD-2 [36] were included (see the Supplementary Materials for further details).

### Data analyses

#### Measurement properties

We used MPlus version 8.7 [37] to explore measurement properties with a latent variable modelling approach. To test the latent structure of each self-report measure we used confirmatory factor analyses with a robust mean and variance adjusted weighted least squares (WLSMV) estimator, with either a model for binary (Yes vs. No responses) or ordered categorical data (questionnaires with multiple ordered response options) depending on the type of responses used for each scale. Because each of the self-report questionnaires which we focus on here have well-established factor structures, we relied on confirmatory factor analyses. We used the root mean square error of approximation (RMSEA, [38]), the comparative fit index (CFI, [39]), and the Tucker-Lewis Index (TLI, [40]) to determine model fit. We interpreted RMSEA values up to 0.05 as indicating good fit, and values up to 0.08 as indicating adequate fit [41]. In the cases of CFI and TLI, we interpreted values greater than 0.90 as indicating adequate, and those greater than 0.95 as indicating good model fit [42].

Finally, we plotted test information functions (TIF) to evaluate the precision of measurement of the self-report questionnaires using MPlus version 8.7 [37]. TIF plots illustrate Fischer information - i.e., an indicator of the precision or reliability of the measure due to their inverse relationship with the standard error of measurement - at different levels of the underlying latent variable [43]. All analyses exploring the properties of the self-report questionnaires were conducted on the complete sample as well as on the young adult subsample.

#### Item reduction

We aimed to optimise two of the eating disorder measures, the EDE-QS and SCOFF, and two of the body dysmorphia measures, the DCQ and WBIS, by shortening them using item response theory. The diagnostic measures, the EDDS and BDDQ, served instead as measures against which we could validate the emerging results. We relied on the factor analyses conducted for the EDE-QS, SCOFF, DCQ, and WBIS to examine their general properties. Our approach was to take a small number of items which load the highest on the underlying factors (i.e., those with the highest discrimination parameter, ideally three items) to create the short scale, while ensuring that the TIF remains as similar as possible to that of the original scale and that internal consistency also remains optimal.

As the measures included in the present study may be used to screen clinical populations, certain items may provide limited information in the general population despite being important in clinical samples. As here we aimed to develop short measures for use in nonclinical samples, we additionally took into consideration the thresholds related to the items. This way, we attempted to avoid the inclusion of any items which may be less informative in the target sample. Where item thresholds were very high, thus resulting in low item endorsement and, subsequently, low variability in a general (not clinical) population like that of MCS, lower item loadings but thresholds closer to the centre of the distribution of latent factor scores were preferred. Unless otherwise noted, the thresholds did not suggest that items should not be retained.

#### Measurement invariance

To determine whether the measurement properties of the scales were equivalent across sex and age groups, we used a measurement invariance testing strategy. To compare ages, we split the sample according to younger adults (18–39 years) and older adults (40 + years), as in the previous analyses. We tested measurement invariance to explore any potential bias within the self-report questionnaires across sexes or age groups caused by measurement error [18, 19, 44, 45]. We conducted the analyses across four groups (sex * age: younger males, older males, younger females, and older females). We used a WLSMV estimator and tested two levels of invariance: configural invariance, without constraining any measurement parameters to be equal across the groups, and scalar invariance, where the items’ loadings as well as their thresholds are constrained to be equal across the groups. We compared the goodness-of-fit indices of the two models. Since the chi-square difference test is very sensitive to sample size, invariance was also informed by additional fit indices. Models where the loss of fit was less than 0.01 for CFI and 0.015 for RMSEA met the criteria for invariance [46, 47]. These analyses were conducted using MPlus version 8.7 [37].

Note that this type of strategy could not be implemented in scales with three or less items, since in those cases the configural model is just-identified at best, thus resulting in non-meaningful goodness-of-fit indices that cannot be compared to those from models with invariance constraints. It was thus not possible to test measurement invariance in the short versions of the scales comprised of only three items. We performed the analyses on the 12- and 5-item EDE-QS, 5-item SCOFF, 7-item DCQ, and 11-item WBIS scales. This allowed us to detect potential differences in the measurement properties of the larger scales that may impact the shorter versions.

#### Scale properties

We first explored scale properties by examining descriptive statistics. To test whether any differences exist in the sample on key measures among sex and age groups (i.e., 18–39 year olds vs. 40 + year olds), we ran independent samples t-tests. We also conducted 2 × 2 ANOVAs to explore any interactions across sex and age groups. The two participants who did not disclose their sex were excluded from the analyses where sex differences were tested. We used SPSS 27.0 to conduct these analyses. We used the Omega macro for SPSS [48] to test the internal consistency of the scales with McDonald’s omega total (*ω*_*t*_) coefficient [49].

#### Correlations

We conducted bivariate correlations between the long and short versions of the eating disorder and body dysmorphia, and between these measures and those of depression, anxiety, and psychological distress. This way, we were able to explore the equivalence in the rank ordering across the measures, along convergent and discriminant validity.

## Results

### Measurement properties & item reduction

**Fig. 1:**
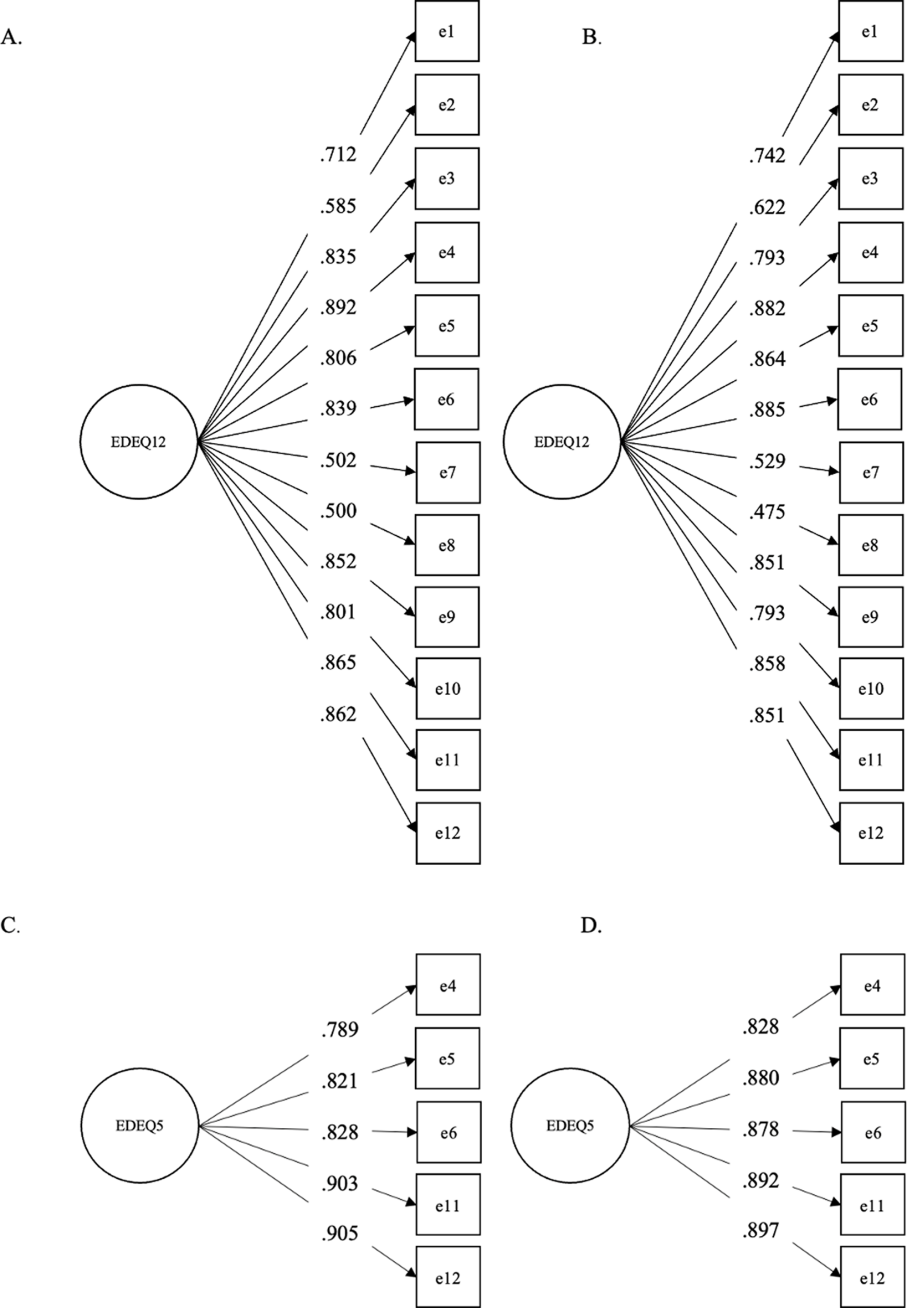
The Results of a Confirmatory Factor Analysis (Standardized Coefficients) on the 12-Item EDE-QS in the (**A**) Full Sample and (**B**) Young Adult Subsample, and on the 5-Item EDE-QS in the (**C**) Full Sample and (**D**) Young Adult Subsample. *Note* The variance of the factors was fixed to 1 in all cases

**Fig. 2:**
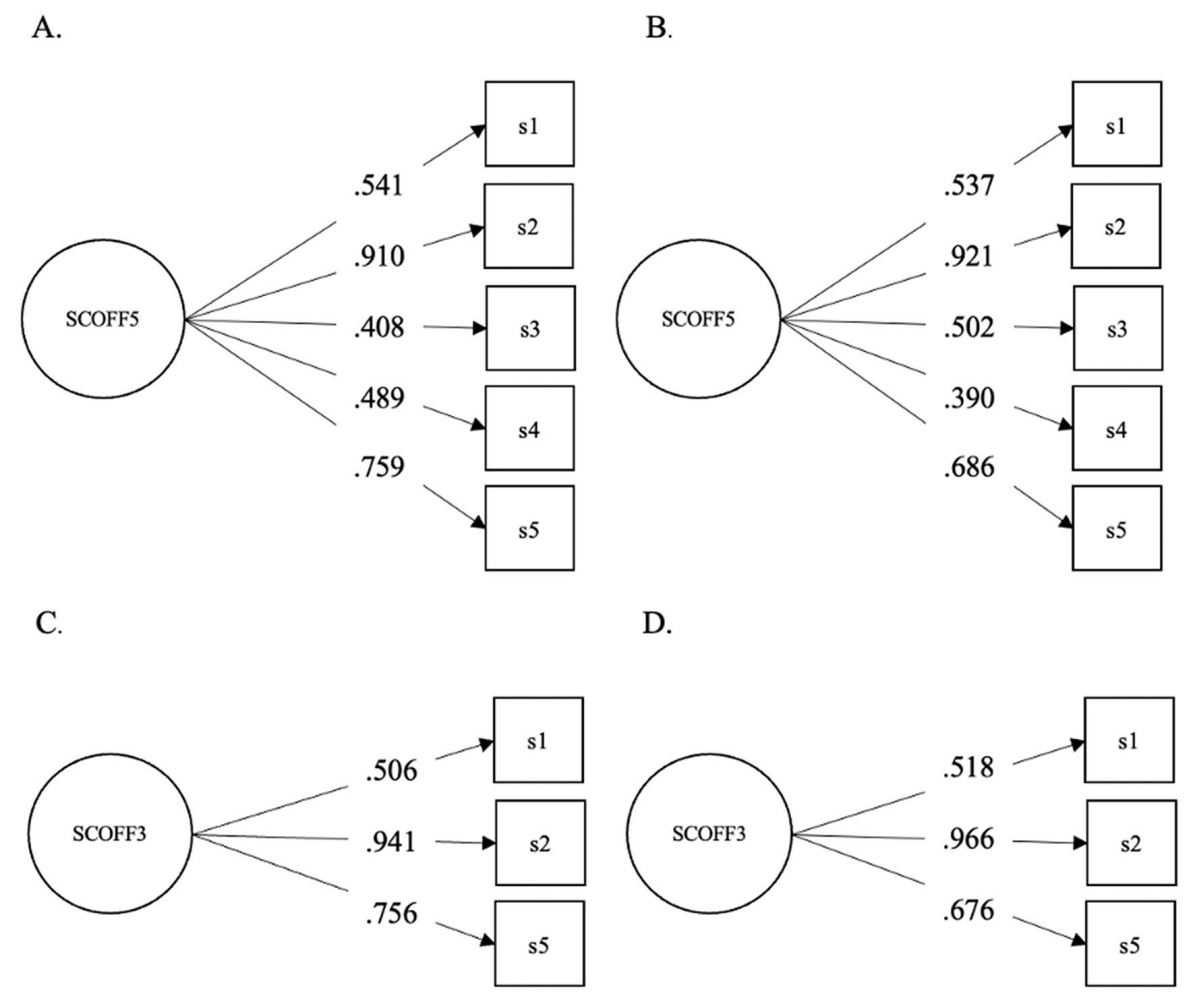
The Results of a Confirmatory Factor Analysis (Standardized Coefficients) on the 5-Item SCOFF in the (**A**) Full Sample and (**B**) Young Adult Subsample, and on the 3-Item SCOFF in the (**C**) Full Sample and (**D**) Young Adult Subsample. *Note* The variance of the factors was fixed to 1 in all cases

**Fig. 3:**
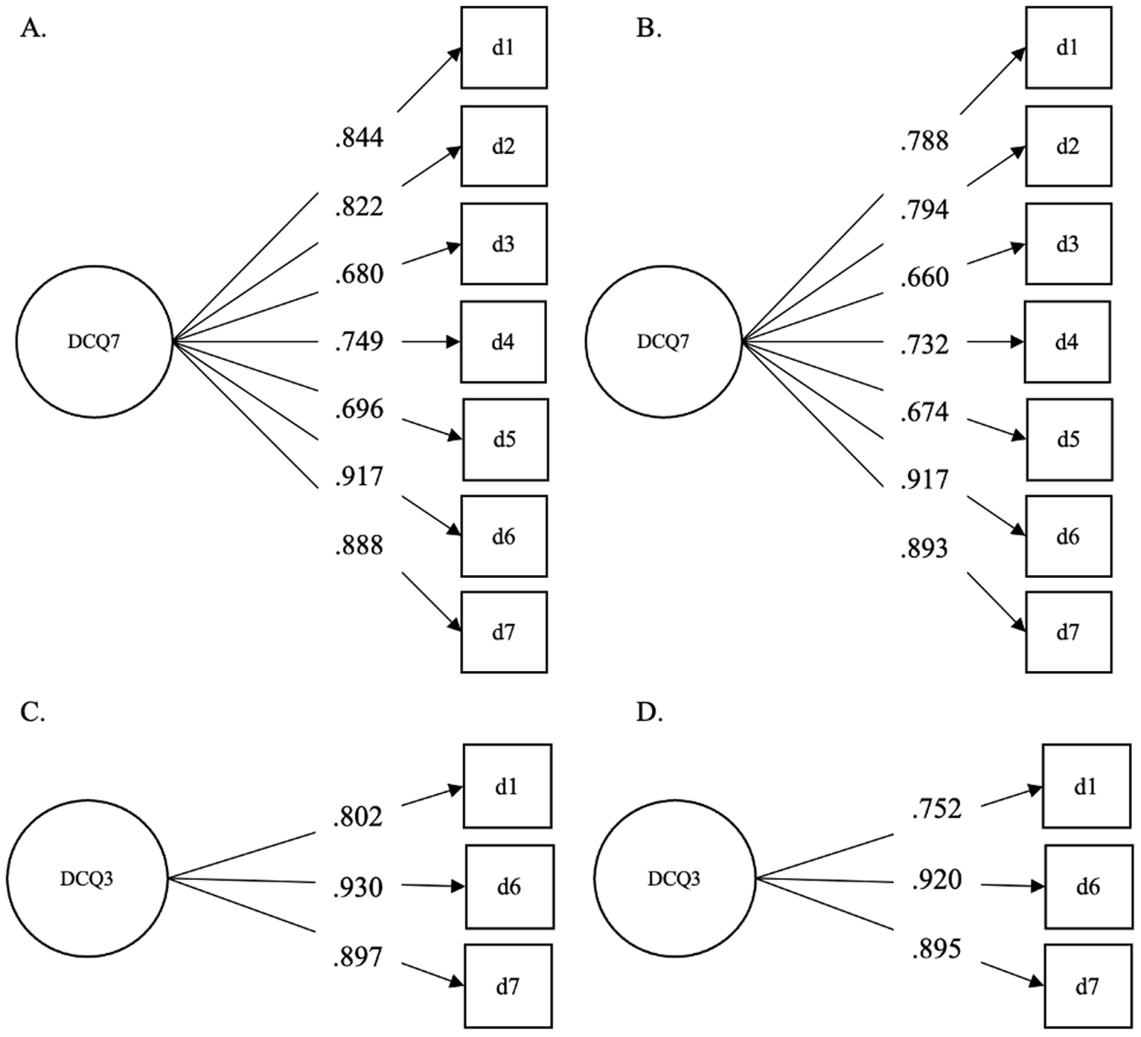
The Results of a Confirmatory Factor Analysis (Standardized Coefficients) on the 7-Item DCQ in the (**A**) Full Sample and (**B**) Young Adult Subsample, and on the 3-Item DCQ in the (**C**) Full Sample and (**D**) Young Adult Subsample. *Note* The variance of the factors was fixed to 1 in all cases

**Fig. 4:**
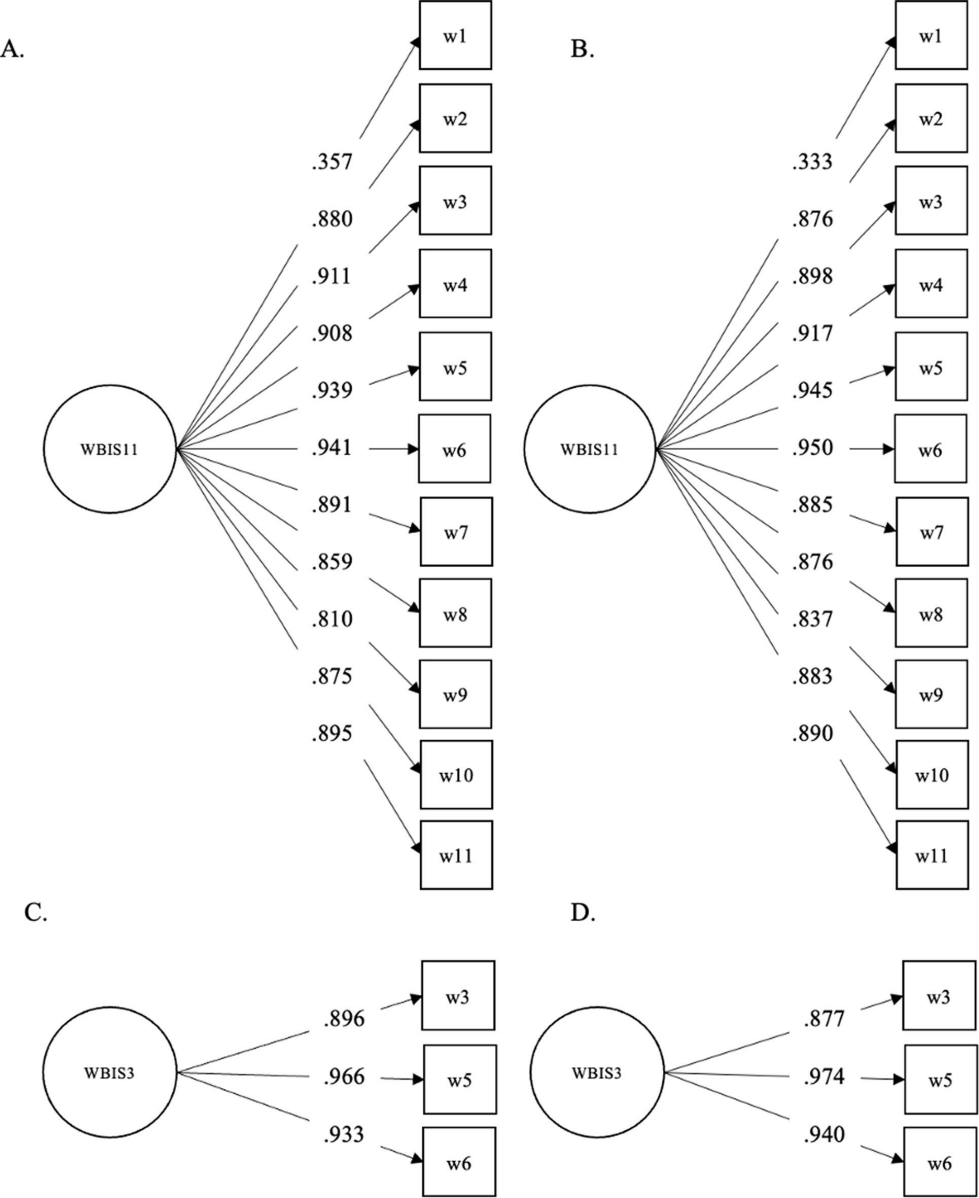
The Results of a Confirmatory Factor Analysis (Standardized Coefficients) on the 11-Item WBIS in the (**A**) Full Sample and (**B**) Young Adult Subsample, and on the 3-Item WBIS in the (**C**) Full Sample and (**D**) Young Adult Subsample. *Note* The variance of the factors was fixed to 1 in all cases

We first conducted a confirmatory factor analysis on the EDE-QS, SCOFF, DCQ, and WBIS scores. Based on these analyses, we created the short versions of the scales, relying on the items with the highest discrimination parameters (Figs. 1, 2, 3 and 4). The fit statistics of the full and shortened administered scales are presented in Table 1, while the TIFs of the scalar models are presented in the Supplementary Materials. While RMSEA values were adequate in the cases of the long and short versions of the SCOFF, the 3-item WBIS, and the 3-item DCQ, the CFI and TLI scores indicated the remaining scales had a good fit as well. The only exception was the 12-item EDE-QS scale assessed in the overall sample rather than the young adult subsample. Nevertheless, this scale also showed an adequate fit.

**Table 1:** Fit statistics for the administered scales

	χ^2^	RMSEA	CFI	TLI	SRMR
EDE-QS-12	1,064.83^***^	0.14^***^	0.94	0.92	0.09
EDE-QS-12Y	449.69^***^	0.14^***^	0.95	0.94	0.08
EDE-QS-5	103.29^***^	0.14^***^	0.99	0.98	0.03
EDE-QS-5Y	57.04^***^	0.17^***^	0.99	0.98	0.03
SCOFF-5	20.75^***^	0.06	0.97	0.95	0.06
SCOFF-5Y	8.84	0.05	0.98	0.96	0.06
SCOFF-3	< 0.001^***^	< 0.001^***^	1.00	1.00	< 0.001
SCOFF-3Y	< 0.001^***^	< 0.001^***^	1.00	1.00	< 0.001
WBIS-11	868.25^***^	0.14^***^	0.99	0.98	0.02
WBIS-11Y	361.53^***^	0.14^***^	0.99	0.98	0.03
WBIS-3	< 0.001^***^	< 0.001^***^	1.00	1.00	< 0.001
WBIS-3Y	< 0.001^***^	< 0.001^***^	1.00	1.00	< 0.001
DCQ-7	126.06^***^	0.09^***^	0.99	0.99	0.03
DCQ-7Y	77.84^***^	0.11^***^	0.98	0.98	0.03
DCQ-3	< 0.001^***^	< 0.001^***^	1.00	1.00	< 0.001
DCQ-3Y	< 0.001^***^	< 0.001^***^	1.00	1.00	< 0.001

Note EDE-QS = Eating Disorder Examination Questionnaire. EDDS-Z = Z transformed eating disorder composite score of the Eating Disorder Diagnostic Scale. EDDS = raw composite score of the Eating Disorder Diagnostic Scale. BDDQ = Body Dysmorphic Disorder Questionnaire. DCQ = Dysmorphic Concern Questionnaire. WBIS = Weight Bias Internalisation Scale. The digits following each label denote the version of the scale described. The letter Y denotes results reflecting only on the young adult (ages 18–39) subsample. ^***^p < .001

#### Eating disorder measures

In the case of the 12-item EDE-QS [10], the three items with the highest loading did not match across the analysis conducted on the full sample and that conducted on the young adult subsample (Fig. 1). Our aim throughout the study was to develop short scales which are optimal for use both in a general UK population as well as the young adult population. This way, test-retest within a single cohort, as well as measurement harmonisation across different UK-based cohorts could be facilitated. For this reason, we chose items with the three highest loadings from both analyses, resulting in a five-item long scale. The final items were *‘On how many of the past 7 days has thinking about your weight or shape made it very difficult to concentrate on things you are interested in (such as working*,* following a conversation or reading)?’. ‘On how many of the past 7 days have you had a definite fear that you might gain weight?’*,* ‘On how many of the past 7 days have you had a strong desire to lose weight?’*,* ‘Over the past 7 days has your weight or shape influenced how you think about (judge) yourself as a person?’*,* ‘Over the past 7 days*,* how dissatisfied have you been with your weight or shape?’* (AppendixA).^3^ These items cover a range of the characteristics of eating disorders, but do not include more clinically salient behaviours such as purging. This indicates that it may be an ideal measure to use among the general, rather than a clinical population.

In the case of the 5-item SCOFF [11], we selected the three items with the highest loadings, which matched across the analysis conducted on the full sample and that conducted on the young adult subsample. These items were *‘Do you make yourself sick because you feel uncomfortably full?’*,* ‘Do you worry you have lost control over how much you eat?’. ‘Would you say that food dominates your life?’* (Fig. 2, Appendix B). Note that the threshold (overall sample: item 1 = 1.63, item 2 = 0.51, item 3 = 0.99, item 4 = 1.09, item 5 = 0.74; young adult sample: item 1 = 1.39, item 2 = 0.34, item 3 = 0.86, item 4 = 0.88, item 5 = 0.61) of item 1 of the SCOFF suggested that though it may hold valuable information in a clinical sample, it may be less useful when assessed in the general population. Indeed, this item was endorsed the least number of times among both the overall sample (only 50 out of 975 participants responded ‘yes’) as well as the young adult sample (only 31 out of 375 participants responded ‘yes’). This corresponds to the content of the item, asking individuals about vomiting on purpose, which may be more applicable to clinical populations. For this reason, we explored a 3-item version of the SCOFF where item 1 was not included. These analyses, however, indicated that when exchanged to the next highest loading item, item 3, its loading in the three-item model was poor (overall sample: item 2 = 0.92, item 3 = 0.32, item 5 = 0.77; young adult sample: item 2 = 0.89, item 3 = 0.44, item 5 = 0.73). We thus retained the 3-item SCOFF which included item 1, despite its seemingly increased suitability for clinical populations.

#### Body dysmorphia measure

In the case of the 7-item DCQ [12], we selected the three items with the highest loadings, which matched across the analysis conducted on the full sample and that conducted on the young adult subsample. These were *‘Have you ever been very concerned about some aspect of your physical appearance?’*,* ‘Have you ever spent a lot of time worrying about a defect in your appearance / bodily functioning?’*,* ‘Have you ever spent a lot of time covering up defects in your appearance / bodily functioning?’* (Fig. 3, AppendixC).

#### Weight bias internalisation measure

A 3-item short version of the 11-item modified WBIS has previously been introduced [13, 14]. The same three items were implicated by our analyses as those with the highest loading when taking the full sample into account. These were *‘I feel anxious about my weight because of what people might think of me’*,* ‘Whenever I think a lot about my weight*,* I feel depressed’*,* ‘I hate myself for my weight’* (Fig. 4, AppendixD). Although when we only included the young adult subsample in the analyses the results did not completely overlap with these, the loadings of the selected three items were nevertheless high. For this reason, and because the three-item version of the scale has already been introduced and used, we decided to keep these items across the remaining analyses.

### Measurement invariance

Across two of the tested questionnaires (the EDE-QS and DCQ), the analyses revealed that none of the older adults (40 + years, either males, females, or both) in the present sample selected the most extreme response options. This could be resolved by grouping the two most extreme categories together among the response options of such items and thus creating an overall cluster with existing responses. However, to form meaningful comparisons, we would in this case have to cluster the responses of the young age group together as well. As the younger age group provided responses across all scales in all categories, this would lead to the loss of information. For the sake of retaining such information, we did not compare the sample across ages, and instead we only explored sex differences within the young adult sample.

Specifically, 0 males over 40 responded with ‘6–7 days’ to the questions ‘On how many of the past 7 days had thinking about food, eating or calories made it very difficult to concentrate on things you are interested in (such as working, following a conversation or reading)?’ (item 3), to ‘On how many of the past 7 days has thinking about your weight or shape made it very difficult to concentrate on things you are interested in (such as working, following a conversation or reading)?’ (item 4), or to ‘On how many of the past 7 days have you tried to control your weight or shape by making yourself sick (vomit) or taking laxatives?’ (item 7) in the 12-item EDE-QS. To item 7, only 1 woman over 40 chose the response ‘6–7 days’, and 0 women over 40 chose the response ‘3–5 days’. Since item 4 was also part of the shortened scale, we again only ran the analyses on the young adult group, testing for bias across sexes. Similarly, in response to the question ‘Have you ever been told by others / doctors that you are normal spite of you strongly believing that something is wrong with your appearance or bodily functioning?’ no women aged over 40 chose the option ‘Much more than other people’, while only a single male over 40 did in the 7-item DCQ. For this reason, we only ran the measurement invariance analysis on the young age group for this scale as well.

Across all remaining measures, we tested measurement invariance across sexes and age groups (i.e., 4 groups: males ages 18–38, females ages 18–39, males ages 40+, females ages 40+). For the sake of consistency, we also conducted all analyses only among the young adult group, comparing the responses of males and females. The results of the measurement invariance testing procedure are presented in Table 2. Whereas RMSEA did not consistently indicate an adequate fit, the changes in the CFI and TLI indicated a good fit across all scales. In the case of the SCOFF scale, the analysis indicated that the residual covariance matrix was not positive definite when conducted on the full sample. Thus, the corresponding results should not be interpreted as the solution may not be valid. The binary nature of the items and the reduced sample sizes resulting from the multiple group approach likely led to estimation issues.

**Table 2:** Measurement invariance testing

	Configural Model					Scalar Model					Differences		
	χ^2^	RMSEA	CFI	TLI	SRMR	χ^2^	RMSEA	CFI	TLI	SRMR	χ^2^	RMSEA	CFI
EDE-QS-12Y	495.10^***^	0.14^***^	0.95	0.93	0.09	508.16^***^	0.12^***^	0.95	0.95	0.09	83.38^***^	0.02	< 0.01
EDE-QS-5Y	65.61^***^	0.17^***^	0.99	0.97	0.04	82.06^***^	0.12^***^	0.99	0.99	0.05	28.91	0.05	< 0.01
SCOFF-5	29.06	0.04	0.98	0.97	0.07	49.69^**^	0.05	0.96	0.94	0.09	19.52^*^	-0.01	-0.02
SCOFF-5Y	9.39	< 0.001	1.00	1.00	0.06	15.12	0.03	0.99	0.98	0.07	4.96	0.03	-0.02
WBIS-11	928.59^***^	0.13^***^	0.99	0.98	0.03	831.65^***^	0.07^***^	0.99	0.995	0.03	258.54^***^	0.06	< 0.01
WBIS-11Y	395.02^***^	0.14^***^	0.99	0.98	0.03	322.12^***^	0.08^***^	0.99	0.99	0.04	62.63	0.06	< 0.01
DCQ-7Y	91.64^***^	0.11^***^	0.98	0.97	0.04	102.12^***^	0.08^*^	0.99	0.99	0.05	27.41	0.03	0.01

*Note* EDE-QS = Eating Disorder Examination Questionnaire. WBIS = Weight Bias Internalisation Scale. DCQ = Dysmorphic Concern Questionnaire. The digits following each label denote the version of the scale described. The letter Y denotes results reflecting only on the young adult (ages 18–39) subsample ^***^p ≤ .001. ^**^p < .01. ^*^p < .05

### Scale properties

Descriptive statistics are presented in Table 3. McDonald’s *ω*_*t*_ suggests that internal consistency remained comparable after removing items from each scale. Note, however, that the omega total values for the SCOFF scale are lower than expected. This likely means that this scale is not measuring a unidimensional construct but rather picks on different things that are not extremely related to each other on average. This is true for both the 5-item scale as well as the 3-item scale, while the 3-item version actually has slightly higher omega values.

**Table 3:** Descriptive statistics of the original and shortened scales

	Full Sample				Young Adult Subsample (Ages 18–39)			
	*M*	*SD*	Range	McDonald’s *ω*_*t*_	*M*	*SD*	Range	McDonald’s *ω*_*t*_
EDE-QS-12	7.07	6.56	0–36	0.90	8.47	7.36	0–36	0.91
EDE-QS-5	4.40	3.93	0–15	0.88	5.06	4.32	0–15	0.90
SCOFF-5	0.89	1.12	0–5	0.61	1.10	1.22	0–5	0.60
SCOFF-3	0.59	0.83	0–3	0.65	0.72	0.89	0–3	0.63
EDDS	17.57	14.30	0–81	-	20.35	15.48	0–81	-
EDDS-Z	-0.004	0.62	-0.69-3.05	-	0.12	0.66	-0.69-3.05	-
BDDQ	0.19	0.39	0 or 1	-	0.29	0.45	0 or 1	-
WBIS-11	37.00	17.48	11–77	0.96	38.78	18.28	11–77	0.96
WBIS-3	9.88	5.58	3–21	0.93	10.65	5.85	3–21	0.92
DCQ-7	5.49	4.46	0–21	0.89	7.04	4.91	0–21	0.88
DCQ-3	2.93	2.27	0–9	0.86	3.66	2.44	0–9	0.86

*Note* EDE-QS = Eating Disorder Examination Questionnaire. EDDS-Z = Z transformed eating disorder composite score of the Eating Disorder Diagnostic Scale. EDDS = raw composite score of the Eating Disorder Diagnostic Scale. BDDQ = Body Dysmorphic Disorder Questionnaire. WBIS = Weight Bias Internalisation Scale. DCQ = Dysmorphic Concern Questionnaire. The digits following each label denote the version of the scale described (i.e., number of items)

Independent samples t-tests revealed that females scored significantly higher than males on all eating disorder and body dysmorphia measures in the overall sample (Table 4A) and in the young adult sample (Table 4B). Younger adults (ages 18–39) also scored significantly higher on all measures than older adults (ages 40+, Table 4C). 2 × 2 ANOVAs revealed a significant interaction between sex and age on the EDDS, BDDQ, and DCQ measures (Table 5). Specifically, the results revealed that the while women consistently scored higher than males across all scales and both age groups, this difference was greater among young adults than older adults. The results on all further measures (apart from the Z-transformed EDDS scores) were also in the same directions (Table 5). The measures of effect size are also included along the results of the t-tests (Cohen’s *d*) and ANOVAs (η_p_^2^).

**Table 4:** Mean comparisons on all assessed measures Across (A) sexes in the overall sample, (B) sexes in the Young Adult Sample, and (C) age groups

**A.**	**Mean Comparisons Across Sexes in the Overall Sample**						
	Females: *M (SD)*	Males: *M (SD)*	*t*	*M* difference *(SE)*	95% CI of difference	*p*	Cohen’s *d*
EDE-QS-12^*^	8.52 (6.89)	5.44 (5.74)	-7.57	-3.08 (0.41)	[-3.88, -2.28]	< 0.001	− 0.48
EDE-QS-5^*^	5.50 (4.13)	3.18 (3.31)	-9.69	-2.32 (0.24)	[-2.79, -1.85]	< 0.001	− 0.62
SCOFF-5^*^	1.10 (1.18)	0.65 (0.98)	-6.38	-0.44 (0.07)	[-0.58, -0.31]	< 0.001	− 0.41
SCOFF-3^*^	0.78 (0.91)	0.38 (0.68)	-7.72	-0.40 (0.05)	[-0.50, -0.30]	< 0.001	− 0.49
EDDS-Z^*^	0.14 (0.66)	-0.16 (0.54)	-7.75	-0.30 (0.04)	[-0.37, -0.22]	< 0.001	− 0.50
EDDS^*^	21.61 (15.02)	13.09 (12.01)	-9.79	-8.53 (0.87)	[-10.23, -6.82]	< 0.001	− 0.62
BDDQ^*^	0.27 (0.44)	0.10 (0.30)	-6.96	-0.17 (0.02)	[-0.22, -0.12]	< 0.001	− 0.44
DCQ-7^*^	6.16 (4.70)	4.71 (4.05)	-5.14	-1.45 (0.28)	[-2.00, -0.89]	< 0.001	− 0.33
DCQ-3^*^	3.35 (2.36)	2.47 (2.08)	-6.12	-0.87 (0.14)	[-1.15, -0.59]	< 0.001	− 0.39
WBIS-11^*^	41.73 (17.95)	31.78 (15.32)	-9.29	-9.95 (1.07)	[-12.05, -7.85]	< 0.001	− 0.59
WBIS-3^*^	11.48 (5.65)	8.13 (4.95)	-9.84	-3.35 (0.34)	[-4.02, -2.68]	< 0.001	− 0.63
**B.**	**Mean Comparisons Across Sexes in the Young Adult Subsample**						
	Females: *M (SD)*	Males: *M (SD)*	*t*	*M* difference *(SE)*	95% CI of difference	*p*	Cohen’s *d*
EDE-QS-12^*^	10.44 (7.58)	6.42 (6.54)	-5.51	-4.02 (0.73)	[-5.46, -2.59]	< 0.001	− 0.57
EDE-QS-5^*^	6.53 (4.34)	3.53 (3.74)	-7.17	-3.00 (0.42)	[-3.82, --2.17]	< 0.001	− 0.74
SCOFF-5^*^	1.40 (1.23)	0.79 (1.12)	-5.02	-0.61 (0.12)	[-0.85, -0.37]	< 0.001	− 0.52
SCOFF-3^*^	0.96 (0.95)	0.47 (0.75)	-5.49	-0.49 (0.09)	[-0.66, -0.31]	< 0.001	− 0.57
EDDS-Z^*^	0.29 (0.68)	-0.06 (0.59)	-5.45	-0.36 (0.07)	[-0.48, -0.23]	< 0.001	− 0.56
EDDS^*^	25.64 (15.70)	14.86 (13.19)	-7.21	-10.78 (1.50)	[-13.72, -7.84]	< 0.001	− 0.74
BDDQ^*^	0.41 (0.49)	0.17 (0.38)	-5.32	-0.24 (0.05)	[-0.33, -0.15]	< 0.001	− 0.55
DCQ-7^*^	8.11 (5.05)	5.93 (4.50)	-4.41	-2.18 (0.49)	[-3.15, -1.20]	< 0.001	− 0.45
DCQ-3^*^	4.23 (2.48)	3.07 (2.25)	-4.75	-1.16 (0.24)	[-1.64, -0.68]	< 0.001	− 0.49
WBIS-11	44.95 (18.11)	32.35 (16.16)	-7.10	-12.59 (1.77)	[-16.08, -9.11]	< 0.001	− 0.73
WBIS-3	12.68 (5.67)	8.55 (5.28)	-7.30	-4.13 (0.57)	[-5.25, -3.02]	< 0.001	− 0.75
**C.**	**Mean Comparisons Across Younger (Ages 18–39) and Older Adults (Ages 40+)**						
	YA: *M (SD)*	OA: *M (SD)*	*t*	*M* difference *(SE)*	95% CI of difference	*p*	Cohen’s *d*
EDE-QS-12^*^	8.47 (7.36)	6.17 (5.83)	5.11	2.29 (0.45)	[1.41, 3.17]	< 0.001	0.36
EDE-QS-5^*^	5.06 (4.32)	3.97 (3.61)	4.05	1.08 (0.27)	[0.56, 1.61]	< 0.001	0.28
SCOFF-5^*^	1.10 (1.22)	0.75 (1.03)	4.69	0.35 (0.08)	[0.21, 0.50]	< 0.001	0.32
SCOFF-3^*^	0.72 (0.89)	0.50 (0.78)	3.85	0.22 (0.06)	[0.11, 0.33]	< 0.001	0.26
EDDS-Z^*^	0.12 (0.66)	-0.08 (0.58)	4.83	0.20 (0.04)	[0.12, 0.28]	< 0.001	0.33
EDDS^*^	20.35 (15.48)	15.80 (13.23)	4.71	4.55 (0.97)	[2.66, 6.45]	< 0.001	0.32
BDDQ^*^	0.29 (0.45)	0.12 (0.33)	6.21	0.17 (0.03)	[0.11, 0.22]	< 0.001	0.44
DCQ-7^*^	7.04 (4.91)	4.49 (3.84)	8.55	2.55 (0.30)	[1.96, 3.14]	< 0.001	0.60
DCQ-3^*^	3.66 (2.44)	2.48 (2.04)	7.81	1.18 (0.15)	[0.88, 1.48]	< 0.001	0.54
WBIS-11	38.77 (18.28)	35.90 (16.85)	2.50	2.87 (1.15)	[0.62, 5.13]	0.01	0.17
WBIS-3^*^	10.65 (5.85)	9.40 (5.35)	3.35	1.24 (0.37)	[0.53, 1.97]	0.001	0.23

*Note* EDE-QS = Eating Disorder Examination Questionnaire. EDDS-Z = Z transformed eating disorder composite score of the Eating Disorder Diagnostic Scale. EDDS = raw composite score of the Eating Disorder Diagnostic Scale. BDDQ = Body Dysmorphic Disorder Questionnaire. DCQ = Dysmorphic Concern Questionnaire. WBIS = Weight Bias Internalisation Scale. The digits following each label denote the version of the scale described (i.e., number of items).^*^denotes analyses where Levene’s test for equality of variances was significant, so the presented results are adjusted for equal variances not being assumed

**Table 5:** Interactions between sex and age on all assessed measures

	F	df	*p*	η_*p*_^2^
EDE-QS-12	3.13	1	0.08	0.003
EDE-QS-5	4.73	1	0.03	0.01
SCOFF-5	3.30	1	0.07	0.003
SCOFF-3	1.73	1	0.19	0.002
EDDS-Z	1.26	1	0.26	0.001
EDDS	3.96	1	0.047	0.004
BDDQ	5.24	1	0.02	0.01
DCQ-7	4.00	1	0.046	0.004
DCQ-3	2.29	1	0.13	0.002
WBIS-11	3.69	1	0.06	0.004
WBIS-3	3.13	1	0.08	0.003

*Note* EDE-QS = Eating Disorder Examination Questionnaire. EDDS-Z = Z transformed eating disorder composite score of the Eating Disorder Diagnostic Scale. EDDS = raw composite score of the Eating Disorder Diagnostic Scale. BDDQ = Body Dysmorphic Disorder Questionnaire. DCQ = Dysmorphic Concern Questionnaire. WBIS = Weight Bias Internalisation Scale. The digits following each label denote the version of the scale described (i.e., number of items)

### Correlations

Scores from all eating disorder, body dysmorphia, and weight bias internalisation scales were positively correlated with each other in the overall sample as well as the young adult subsample (*r*s ranging from 0.40 to 0.96; Table 6). The short scales corresponding to their longer versions showed the strongest positive correlations (*r*s ranging from 0.88 to 0.96), as expected. While the data we collected on depression, anxiety, and psychological distress are presented in detail elsewhere [9], bivariate correlations revealed that all these measures were significantly positively correlated to those assessing eating disorders, body dysmorphia, and weight bias internalisation (*r*s ranging from 0.32 to 0.57). Lower psychological distress, lower depression, and lower anxiety were related to lower levels of eating disorders, lower levels of body dysmorphia, and lower levels of weight bias internalisation. These findings are presented on OSF (due to the large size of the correlation table, https://osf.io/vg4a9/). Despite the significant correlations across all measures, the pattern of correlations among scales designed to assess more closely related concepts (i.e., eating disorders with body dysmorphia and weight bias internalisation; depression with anxiety and psychological distress) are stronger with each other. These results further support the discriminant and convergent validity of the scales presented here.

**Table 6:** Correlations among the eating disorder and body dysmorphia measures in the overall sample and young adult sample

	1. EDE-QS-12	2. EDE-QS-5	3. SCOFF	4. SCOFF-3	5. EDDS-Z	6. EDDS	7. BDDQ	8. DCQ-7	9. DCQ-3	10. WBIS-11
1. EDE-QS-12	-									
2. EDE-QS-5	0.95^***^ / 0.94^***^	-								
3. SCOFF	0.60^***^ / 0.58^***^	0.56^***^ / 0.55^***^	-							
4. SCOFF-3	0.60^***^ / 0.57^***^	0.57^***^ / 0.54^***^	0.88^***^ / 0.88^***^	-						
5. EDDS-Z	0.77^***^ / 0.77^***^	0.73^***^ / 0.72^***^	0.62^***^ / 0.57^***^	0.65^***^ / 0.59^***^	-					
6. EDDS	0.82^***^ / 0.83^***^	0.80^***^ / 0.81^***^	0.61^***^ / 0.58^***^	0.63^***^ / 0.59^***^	0.95^***^ / 0.95^***^	-				
7. BDDQ	0.53^***^ / 0.55^***^	0.54^***^ / 0.57^***^	0.40^***^ / 0.42^***^	0.41^***^ / 0.43^***^	0.45^***^ / 0.47^***^	0.48^***^ / 0.50^***^	-			
8. DCQ-7	0.54^***^ / 0.51^***^	0.56^***^ / 0.54^***^	0.43^***^ / 0.40^***^	0.42^***^ / 0.40^***^	0.51^***^ / 0.49^***^	0.55^***^ / 0.52^***^	0.51^***^ / 0.51^***^	-		
9. DCQ-3	0.55^***^ / 0.53^***^	0.57^***^ / 0.56^***^	0.45^***^ / 0.43^***^	0.43^***^ / 0.42^***^	0.50^***^ / 0.49^***^	0.56^***^ / 0.53^***^	0.51^***^ / 0.52^***^	0.95^***^ / .95^***^	-	
10. WBIS-11	0.72^***^ / 0.74^***^	0.79^***^ / 0.82^***^	0.52^***^ / 0.49^***^	0.56^***^ / 0.52^***^	0.70^***^ / 0.68^***^	0.78^***^ / 0.76^***^	0.49^***^ / 0.51^***^	0.57^***^ / 0.56^***^	0.58^***^ / 0.59^***^	-
11. WBIS-3	0.71^***^ / 0.74^***^	0.78^***^ / 0.81^***^	0.52^***^ / 0.49^***^	0.55^***^ / 0.51^***^	0.70^***^ / 0.66^***^	0.77^***^ / 0.75^***^	0.49^***^ / 0.51^***^	0.56^***^ / 0.54^***^	0.58^***^ / 0.57^***^	0.96^***^ / 0.96^***^

*Note* EDE-QS = Eating Disorder Examination Questionnaire. EDDS-Z = Z transformed eating disorder composite score of the Eating Disorder Diagnostic Scale. EDDS = raw composite score of the Eating Disorder Diagnostic Scale. BDDQ = Body Dysmorphic Disorder Questionnaire. DCQ = Dysmorphic Concern Questionnaire. WBIS = Weight Bias Internalisation Scale. The digits at following each label denote the version of the scale described.Correlations among the full sample are presented in **bold.** Correlations among the young adult sample are presented in italics.^***^p < .001

## Discussion

Throughout the analyses presented in this manuscript, we developed short versions of existing, widely used measures of eating disorders and body dysmorphia. Specifically, we aimed to identify short sets of items which capture similar information and variance as do the full scales, and which correlate well with the full scales, but can be completed in less time. While using shorter measures may optimise data quality across research settings due to eliminating unnecessary confounds as fatigue and boredom among research participants [2], brevity may be especially important when it comes to asking participants about sensitive topics such as behaviours related to eating disorders [15–17]. Based on the analyses, we introduce here a 5-item short version of the 12-item EDE-QS [10], a 3-item short version of the 5-item SCOFF [11], and a 3-item short version of the 7-item DCQ [12]. We further explored the properties of the 11-item WBIS [14], a measure of weight bias internalisation, with our results supporting the validity of its recently introduced 3-item version [13].

The short version of each of the scales correlated strongly positively with their longer versions. Furthermore, the scales correlated positively as expected with alternative measures of eating disorders and body dysmorphia, though these correlations were somewhat weaker. Finally, all measures also correlated with measures of psychological distress, depression, and anxiety, but these correlations, although significant, were the weakest among those observed. As expected, these revealed that an increased presence of eating disorder, body dysmorphia, and weight bias internalisation symptoms were related to increased psychological distress, depression, and anxiety. These findings support the convergent and discriminant validity of the measures.

The short scales performed similarly to the longer versions across additional analyses. The results corroborated previous findings indicating a greater prevalence of eating disorders and body image concerns among females than males [50–52] and among younger compared to older individuals [53–55]. Consistently, the difference between men and women was greater in the young adult group then in the older adult group, although this interaction did not reach significance across all analyses.

We observed measurement invariance across age and sex groups in the 11-item WBIS scale and across sex groups among young adults in the 5-item SCOFF, 12- and 5-item EDE-QS, 11-item WBIS, and 7-item DCQ. This could not be formally tested across the scales with 3 or fewer items. We also refrained from testing it across the full sample on the 12- and 5-item EDE-QS and the 7-item DCQ. This was because on some items, cases existed where no older adults endorsed extreme response options. For example, 0 males over 40 and only 1 woman over 40 responded with the option ‘6–7 days’ to the question *On how many of the past 7 days have you tried to control your weight or shape by making yourself sick (vomit) or taking laxatives?* This likely reflects [1] the observed difference across age groups, indicating that indeed younger adults have a greater likelihood of experiencing symptoms of eating disorders and body dysmorphia and [2] that the data was collected among a nonclinical sample of adults where more extreme symptoms are rare. We chose not to conduct measurement invariance testing on these scales as in order to do so, we would have merged some response options together to ensure that each one has some participants endorsing it. To be able to compare the responses of older and younger adults, we would have had to also merge across the responses of younger adults, where such an issue with a lack of participants selecting a response option was not present, ultimately leading to a loss of information. Furthermore, the results from the 5-item SCOFF scale could not be interpreted across the full sample, likely caused by the binary responses and the reduced sample sizes caused by splitting across age and sex groups. Nevertheless, the lack of issues in at least one version of each scale, as indicated by measurement invariance testing, may suggest that the corresponding alternative versions of the same scales may also have invariant measurement properties across the same groups. The analyses further suggest that the short scales may provide a good approximation of the longer versions. This is also supported by the high loadings of the items of the short scales on the underlying latent variables.

The analyses presented here were limited by the nature of the short scales. Some of the analyses presented on the full scales could not be conducted on the short scales due to the number of items included. For example, measurement invariance testing cannot be implemented in scales with three or less items. In addition, the present analyses were conducted in a sample of UK adults. Based on the results presented here, we cannot be certain whether they would replicate in different cultural or national contexts. Finally, it should be noted that the results presented here were collected from the general population. We thus cannot make any conclusions based on these results about the performance of the scales in clinical populations.

As such, we urge future research to investigate the short measures presented here among clinical samples to further explore the contexts in which they may be used. It would further be desirable to test the performance of the short scales among groups known to differ in eating disorders and related pathologies from the general population, including lesbian, gay, bisexual, and transgender individuals [56] or gender-expansive individuals who identify outside the binary system of man or woman [57] and assess whether the differences across such groups established through the use of alternative measures replicates. Such results would further support the construct validity of the short measures.

## Conclusions

This manuscript presents the short versions of eating disorder and body dysmorphia measures, specifically of the 12-item EDE-QS, the 5-item SCOFF, and the 7-item DCQ. It further investigates the properties of the already established 3-item version of the 11-item WBIS, a measure of weight bias internalisation. The analyses indicate that these short measures may perform comparably to their longer versions. The short measures may prove invaluable to research where the amount of time spent on any given measure is scarce, including cohort studies. Across research involving questionnaire-type measures, brevity may contribute to data quality as it eases response burden, and reduces the likelihood of participants experiencing fatigue or boredom effects.

## Appendix A

Eating Disorder Examination Questionnaire [10].

12-item scale:

On how many of the past 7 days….

1. Have you been deliberately trying to limit the amount of food you eat to influence your weight or shape (whether or not you have succeeded)?
2. Have you gone for long periods of time (e.g., 8 or more waking hours) without eating anything at all in order to influence your weight or shape?
3. Has thinking about food, eating or calories made it very difficult to concentrate on things you are interested in (such as working, following a conversation or reading)?
4. **Has thinking about your weight or shape made it very difficult to concentrate on things you are interested in (such as working**,** following a conversation or reading)?**
5. **Have you had a definite fear that you might gain weight?**
6. **Have you had a strong desire to lose weight?**
7. Have you tried to control your weight or shape by making yourself sick (vomit) or taking laxatives?
8. Have you exercised in a driven or compulsive way as a mean of controlling your weight, shape or body fat, or to burn off calories?
9. Have you had a sense of having lost control over your eating (at the time that you were eating)?
10. On how many of these days (i.e., days on which you had a sense of having lost control over your eating) did you eat what other people would regard as an unusually large amount of food in one go?

Over the past 7 days….

11. ** Has your weight or shape influenced how you think about (judge) yourself as a person?**
12. ** How dissatisfied have you been with your weight or shape?**

Responses: Items 1–10: 0 days, 1–2 days, 3–5 days, 6–7 days; Items 11–12: Not at all, Slightly, Moderately, Markedly.

5-item scale: 4, 5, 6, 11, 12.

## Appendix B

SCOFF [11].

5-item scale:

1. **Do you make yourself sick because you feel uncomfortably full?**
2. **Do you worry you have lost control over how much you eat?**
3. Have you recently lost more than one stone in a three month period?
4. Do you believe yourself to be fat when others say you are too thin?
5. **Would you say that food dominates your life?**

Responses: Yes/No.

3-item scale: 1, 2, 5.

## Appendix C

Dysmorphic Concern Questionnaire [12].

7-item scale:

Have you ever:

1. **Been very concerned about some aspect of your physical appearance?**
2. Considered yourself to be misinformed or misshaped in some way (e.g., nose / hair / skin / sexual organ / overall body build)?
3. Considered your body to be malfunctional in some way (e.g., excessive body odour, flatulence, sweating)?
4. Considered or felt that you needed to consult a plastic surgeon / dermatologist / physician about these concerns?
5. Been told by others / doctors that you are normal spite of you strongly believing that something is wrong with your appearance or bodily functioning?
6. **Spent a lot of time worrying about a defect in your appearance / bodily functioning?**
7. **Spent a lot of time covering up defects in your appearance / bodily functioning?**

Responses: Not at all, Same as other people, More than most people, Much more than most people.

3-item scale: 1, 6, 7.

## Appendix D

Modified Weight Bias Internalisation Scale [14].

11-item scale:

1. Because of my weight, I feel that I am just as competent as anyone.
2. I am less attractive than most people because of my weight.
3. **I feel anxious about my weight because of what people might think of me.**
4. I wish I could drastically change my weight.
5. **Whenever I think a lot about my weight**,** I feel depressed.**
6. **I hate myself for my weight.**
7. My weight is a major way that I judge my value as a person.
8. I don’t feel that I deserve to have a really fulfilling social life, because of my weight.
9. I am OK being the weight that I am.
10. Because of my weight, I don’t feel like my true self.
11. Because of my weight, I don’t understand how anyone attractive would want to date me.

Responses: 7-point Likert scale, 1 = Strongly disagree, 7 = Strongly agree.

3-item scale [13]: 3, 5, 6.

Note: items 1& 9 are reverse scored.

## Electronic supplementary material

Below is the link to the electronic supplementary material.

Supplementary Material 1

## Data Availability

All data and corresponding syntax files are available via OSF: https://osf.io/vg4a9/.

## Footnotes

1 Note that the preregistration did not include the plan to test measurement invariance.

2 To indicate their sex, participants were asked to respond to the question *‘Which of the following were you described as at birth?’* by selecting one of the following options: *male*,* female*,* intersex*,* prefer not to say.*

3 Note that item 9 of the EDE-QS may have performed well within the short scale, as indicated by the factor loadings. Due to human error, we overlooked this when conducting the analyses. The short questionnaire has been included in the Millennium Cohort Study’s 2023 data sweep as described in this manuscript. The near perfect correlations between the long and short EDE-QS suggest that regardless of this error, the short version captures an overlapping underlying construct.
