# Supplementary material for: The validation of short eating disorder, body dysmorphia, and Weight Bias Internalisation Scales among UK adults
Source: J Eat Disord. 2024 Sep 9;12:137. doi: 10.1186/s40337-024-01095-9 (PMC11386377; doi:10.1186/s40337-024-01095-9)
Supplement: Supplementary file 1 — Supplementary Material 1 [file 40337_2024_1095_MOESM1_ESM.docx]

**Supplementary Materials**

**The Validation of Short Eating Disorder, Body Dysmorphia, and Weight Bias Internalisation Scales Among UK Adults**

Dorottya Lantos,

Darío Moreno-Agostino,

Lasana T. Harris,

George Ploubidis,

Lucy Haselden,

&

Emla Fitzsimmons

**Measures**

***Psychological distress and anxiety*** were assessed using the 10-item K10 scale and the 6-item K6 scale embedded in it (Kessler et al., 2002), the 9-item version of the Malaise Inventory (Ploubidis et al., 2019; Rutter et al., 1970), the PHQ-9 (Kroenke et al., 2001; Kroenke & Spitzer, 2002), PHQ-2 (Kroenke et al., 2003), GAD-7 (Spitzer et al., 2006), and GAD-2 (Kroenke et al., 2007).

The 10-item K10 and the 6-item K6 scale embedded in it (Kessler et al., 2002) were completed by 971 participants. Participants responded to the items (e.g., During the last 30 days, about how often did you feel hopeless?) on a 5-point Likert scale (1 = none of the time, 5 = all of the time). Participants’ responses were summed, with higher scores indicating greater psychological distress.

The 9-item version of the Malaise Inventory (Ploubidis et al., 2019; Rutter et al., 1970) was completed by 974 participants. Participants completed the items of the questionnaire (e.g., Do you often feel miserable or depressed?) using binary yes/no responses. We scored ‘yes’ responses as 1 and ‘no’ responses as 0, and summed participants’ overall answers, with higher scores indicating greater psychological distress.

The PHQ-9 (Kroenke et al., 2001; Kroenke & Spitzer, 2002) and, embedded within it, the PHQ-2 (Kroenke et al., 2003) were completed by 976 participants. Participants responded to the items (e.g., ‘Over the last 2 weeks, how often have you been bothered by any of the following problems? – Little interest or pleasure in doing things’) on a 4-point Likert scale (0 = not at all, 3 = nearly every day). Participants’ responses were summed, with higher scores indicating increased experiences of depressed mood.

The GAD-7 (Spitzer et al., 2006) and, embedded within it, the GAD-2 (Kroenke et al., 2007) were completed by 974 participants. Participants responded to the items (e.g., ‘Over the last 2 weeks, how often have you been bothered by the following problems? – Feeling nervous, anxious, or on edge’) on a 4-point Likert scale (0 = not at all, 3 = nearly every day). Participants’ responses were summed, with higher scores indicating increased experiences of anxiety.

Total Information Curves of Configural Models of the 5-Item SCOFF, 12- and 5-Item EDE-QS, 11-Item WBIS, and 7-Item DCQ Scales Illustrated Across Sexes and Ages


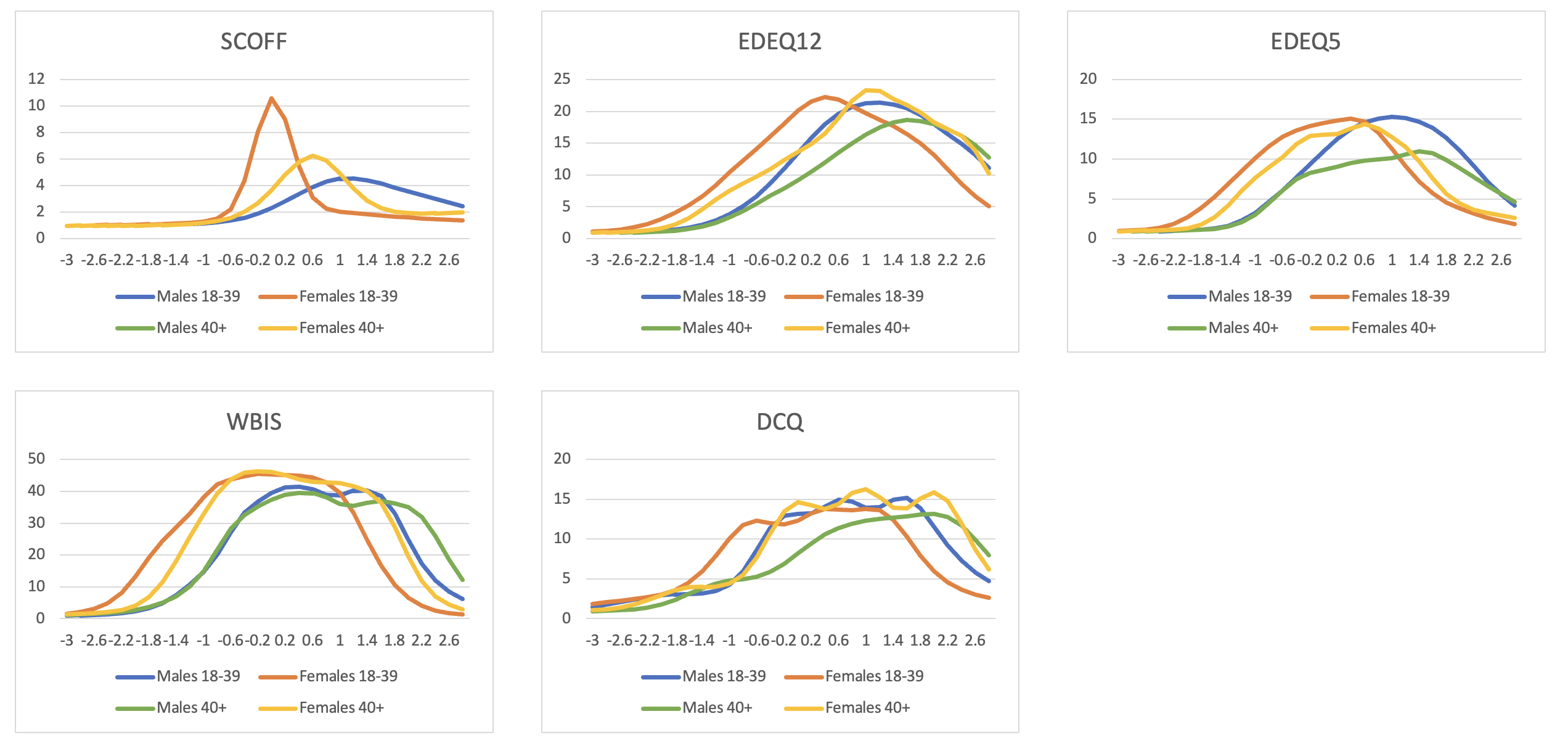


*Note.* The values of the Y-axis represent Fisher information, where higher values indicate greater measurement precision. The values of the X-axis represent the levels of the latent factor (as standard deviations from a mean of 0). The information curve could not be plotted for older males in the case of the SCOFF questionnaire as item 2 did not contribute to the variance here.
